# Pathways linking BMI trajectories and mental health in an adult population-based cohort: role of emotional eating and body dissatisfaction

**DOI:** 10.1038/s41366-025-01772-y

**Published:** 2025-04-07

**Authors:** Stephanie Schrempft, Cecilia Jiménez-Sánchez, Hélène Baysson, María-Eugenia Zaballa, Julien Lamour, Silvia Stringhini, Idris Guessous, Mayssam Nehme, on behalf of the Specchio study group

**Affiliations:** 1 Division of Primary Care Medicine, Unit of Population Epidemiology, Geneva University Hospitals, Chemin Thury 3b, 1206 Geneva, Switzerland; 2 Department of Cell Physiology and Metabolism, Faculty of Medicine, University of Geneva, Geneva, Switzerland; 3 Faculty Diabetes Centre, Faculty of Medicine, University of Geneva, Geneva, Switzerland; 4 Department of Surgery, Division of Thoracic and Endocrine Surgery, Geneva University Hospitals, Geneva, Switzerland; 5 Department of Health and Community Medicine, Faculty of Medicine, University of Geneva, Geneva, Switzerland; 6 School of Population and Public Health, Faculty of Medicine, University of British Columbia, Vancouver, BC Canada; 7 Division of Primary Care Medicine, Geneva University Hospitals, Geneva, Switzerland

**Keywords:** Risk factors, Epidemiology

## Abstract

**Background:**

Overweight and obesity are associated with poor mental health, and the association is bidirectional. Few studies have examined the association between weight change and mental health over time. We aimed to provide further insight into the association between weight gain and mental health, with a focus on emotional eating and body dissatisfaction as mediating factors.

**Methods:**

Height and weight were self-reported upon registration, and in Spring 2022, 2023, and 2024 in the Specchio cohort (Geneva, Switzerland). BMI trajectories were estimated by (1) mixed-effects models to calculate participants’ personal slopes (increase in BMI score per year), and (2) testing the odds of an upward BMI category transition from baseline to last follow-up. The associations of behavioural and psychosocial factors with BMI trajectories (slopes and transitions), and BMI trajectories with mental health outcomes were estimated using regressions adjusted for age, sex, education, and physical health condition. Structural equation modelling was used to test mediating pathways.

**Results:**

Among 7388 participants (59% women, mean age 51 years), factors associated with increasing BMI over 4 years included financial hardship, short sleep duration, less physical activity, more leisure screen time, depressive and anxiety symptoms, and emotional eating (β range [95% CI] = 0.03 [0, 0.05]–0.12 [0.09, 0.15]). Increasing BMI was associated with body dissatisfaction (β = 0.36 [0.33, 0.38]) and poorer quality of life (β = −0.06 [−0.09, −0.03]) at 4-year follow-up after adjustment for anxiety and depressive symptoms at baseline. Emotional eating partly mediated the association between anxiety and depressive symptoms at baseline and increasing BMI, and between financial hardship and increasing BMI. Body dissatisfaction and poorer self-rated health partly mediated the association between increasing BMI and quality of life at follow-up.

**Conclusions:**

Emotional eating and body dissatisfaction contribute to the association between BMI trajectories and mental health and should be considered in weight management and mental health promotion strategies.

## Introduction

Over the past five decades, the prevalence of overweight and obesity has risen throughout the world [1, 2], with global obesity prevalence estimated to reach 20% by 2025 [2]. Overweight and obesity pose substantial risk to health [3], and place enormous strain on the economy [4]. Longitudinal epidemiological research is needed to further understand the role of modifiable risk factors for weight gain, as well as the moderators and underlying mechanisms that link weight gain and health outcomes.

Large-scale population-based epidemiological research has established that overweight and obesity are associated with poor mental health, particularly depression [5–9]. Moreover, the association seems to be bidirectional, with excess weight increasing the risk of depression, and depression increasing the risk of future excess body weight. However, there is little research examining the association between weight change and mental health over time. One recent study involving 2416 Australian participants found that weight gain from childhood to mid-adulthood was associated with a higher risk of adulthood depression and greater symptom severity, but did not have information on prior mental health [10].

Research has identified biological mediators of the association between BMI status and mental health, notably gut microbiota and inflammation [9, 11]. Less research has formally tested behavioural and psychosocial mediators, which may act as higher-level factors, and may be more readily targeted in interventions. Some research has identified emotional eating – defined as eating in response to negative emotions – as a behavioural mechanism linking depression and subsequent excess weight [12], as well as contributing to social disparities in weight [13]. Moreover, the effects of emotional eating on BMI may be mitigated by other health behaviours and traits, such as physical activity [14], sleep [12], and self-regulatory capacity [15]. Other research in adolescents identified poor body image as a psychosocial mechanism linking weight gain and subsequent poor mental health [16], which is yet to be replicated in adults.

In this adult population-based study, we aimed to provide further insight into the association between weight gain and mental health. We examined: (1) a range of behavioural and psychosocial predictors of BMI trajectories (measured as change in BMI score per year, as well as change in BMI category) in adults, including hypothesised interactions between factors, (2) a range of mental health outcomes associated with BMI trajectories, including positive psychology constructs alongside depression and anxiety, and (3) factors contributing to the association between BMI trajectories and mental health, with a focus on emotional eating and body dissatisfaction.

## Materials and methods

### Participants and study design

The data were drawn from Specchio, a population-based digital study launched in December 2020 to follow up COVID-19 serosurvey participants in Geneva, Switzerland [17]. Participants were randomly selected from the Bus Santé population-based study [18], from Geneva population registries [19, 20], or from a list of private and public companies and institutions [21]. Upon registration, participants completed an initial online questionnaire. Subsequent questionnaires were proposed on an annual basis. Participants provided informed consent, and the study was approved by the Cantonal Research Ethics Commission of Geneva, Switzerland (project number 2020–00881). All methods were conducted in accordance with the relevant guidelines and regulations.

Of the 13,260 individuals who completed an inclusion questionnaire, we included those with height and weight data at inclusion and at least one additional time point (*N* = 7429; 56% of the total sample). After cleaning the height, weight, and BMI data, information from 7388 participants was retained for analysis (see supplementary information for a description of the data cleaning procedures).

### Measures

#### BMI

Weight (self-reported) was assessed upon inclusion, and at three subsequent time points (Spring 2022, 2023, and 2024). Height (self-reported) was assessed at registration, and at two subsequent time points (Spring 2023 and 2024). We calculated BMI [weight (kilograms (kg))/height (m)^2^] at each of the four time points based on weight at that time point and the average of the available height values, assuming height did not change during the study period (correlation between ‘average’ height and its constituent height values was 0.99). We also classified participants into BMI categories at each time point using the World Health Organisation guidelines: underweight (BMI < 18.5 kg/metres (m)^2^), healthy weight (BMI 18.5–24.9 kg/m^2^), overweight (BMI 25.0–29.9 kg/m^2^), obesity (BMI 30–39.9 kg/m^2^), and severe obesity (BMI ≥ 40 kg/m^2^). We assessed the validity of self-reported against measured height, weight, and BMI, with good results (see supplementary information).

#### Behavioural and psychosocial factors

Behavioural and psychosocial factors were assessed by questionnaire at inclusion or follow-up (see supplementary Table 1 for an overview). Behavioural factors included moderate and vigorous physical activity, fruit and vegetable consumption, binge drinking, sleep duration, total leisure screen time (hours per day), anti-depressant medication use (yes/no), and emotional eating. Frequency of moderate and vigorous activity were measured on a 6-point scale, and reverse scored to range from 1 (every day) to 6 (never). Fruit and vegetable consumption (portions per day) were assessed using items from the Prevention with Mediterranean Diet assessment tool [22], which were summed and reverse scored. Binge drinking was consuming six or more alcoholic beverages on the same occasion at least once per month (question from The Alcohol Use Disorders Identification Test-Concise). Sleep duration was the average number of hours participants slept at night during week and weekend days. Short sleep duration was < 7 h [23]. Emotional eating was assessed using the 5-item emotional overeating subscale from the Adult Eating Behaviour Questionnaire [24]. Items (e.g., I eat more when I’m upset) are scored on a 5-point Likert scale ranging from 1 strongly disagree to 5 strongly agree (Cronbach’s alpha = 0.95). We did not include smoking cessation as only 157 participants stopped smoking during the study period.

Psychosocial factors included social resources (perceived social support) and risks (perceived financial hardship), as well as psychological resources (self-esteem, self-efficacy, personal mastery) and risks (loneliness, anxiety and depressive symptoms, and perceived stress). Perceived social support was measured using the 3-item Oslo Social Support Scale, with higher scores indicating greater social support (Cronbach’s alpha = 0.60). Financial hardship was facing real financial difficulties to meet ones needs (food, rent, bills, insurance, debt,…), and was coded as no difficulties (‘this has never happened’), average difficulties (‘not now, but in the past’), and important difficulties (‘in the recent past’) [25]. Self-esteem, self-efficacy, and personal mastery were measured using the 5-item Brief Rosenberg Self-Esteem Scale [26], the 4-item form of the Pearlin Mastery Scale [27, 28], and the 3-item General Self-Efficacy Short Scale [29], respectively (Cronbach’s alpha range = 0.82–0.87). Loneliness, depressive and anxiety symptoms, and perceived stress were measured using the 3-item Revised University of California, Los Angeles loneliness scale [30], the 4-item Patient Health Questionnaire [31], and the 10-item Perceived Stress Scale [32], respectively (Cronbach’s alpha range = 0.77–0.88).

#### Mental health outcomes and general health

Mental health outcomes included body satisfaction, well-being, quality of life, as well as depressive and anxiety symptoms measured at follow-up (Spring 2024). Participants rated, on a 10-point scale (1 = not at all satisified, 10 = completely satisfied), the extent to which they were satisfied with their weight and body shape (2 items from the French version of the Body Image State Scale [33]). The body satisfaction score was the mean of the 2 items, which were highly correlated (Pearson’s *r* = 0.86). Subjective well-being was measured using the WHO-5 well-being index, which consists of five statements, each rated on a 6-point scale (0 = never, 5 = all of the time), with higher scores reflecting greater well-being [34]. Internal consistency of the scale was high (Cronbach’s alpha = 0.91). Participants rated their quality of life on a 10-point scale (1 = very poor, 10 = excellent). General self-rated health was assessed on a 5-point scale (0 = very poor, 4 = very good) at inclusion and follow-up (Spring 2024).

#### Covariates

Covariates included age (in years), sex, education level (primary, secondary, or tertiary), and pre-existing physical health condition (yes/no) at baseline.

### Statistical analysis

#### Estimation of BMI trajectories

We examined BMI trajectories in two ways: (1) using mixed-effects models to calculate participants’ personal slopes (change in BMI score per year) across the years of follow-up, and (2) examining the odds of transitioning between BMI categories from baseline to last follow-up.

Mixed-effects models, with a random intercept and a random linear slope, were used to calculate participants’ personal slopes (change in BMI score per year) across the years of follow-up. The models took the form: Bij = (γ0 + γ1ij) + (μ0i + μ1ij) + ϵij, where Bij is the BMI value measured for individual ‘i’ at time ‘j’, γ0 and γ1 are the fixed intercept and slope estimated for the sample, and μ0i and μ1i are the random intercepts and slopes estimated per individual. Each model included years of follow-up as the time indicator, age at baseline, sex, an interaction term between time and age at baseline, and an interaction term between time and sex to properly control for any effect of age at baseline and sex on the rate of BMI change over time. Mixed models do not require an equal number of observations from all participants therefore we included those with BMI data at baseline and at least one additional time point. The random slopes for each participant (annual changes in BMI) were extracted and included in subsequent analyses. We estimated the odds ratios (ORs) of transitioning between BMI categories from baseline to last follow-up using logistic regression models. We focused on those who had an upward BMI classification trajectory (from a healthy BMI to overweight or obesity, from overweight to obesity, or from obesity to severe obesity; 1) compared with those who did not (0).

#### Associations between behavioural and psychosocial factors and BMI trajectories

The associations of behavioural and psychosocial factors with BMI trajectories (slopes and transitions) were estimated using regression analyses (linear and logistic). Each model included age, sex, education, and pre-existing physical health condition at baseline as covariates, alongside the factor of interest. To test whether sex, certain behavioural (physical activity, sleep), and psychosocial (self-efficacy, personal mastery) factors moderated associations between emotional eating and BMI trajectories, we ran a series of regression models, which included the factors of interest (e.g. emotional eating and physical activity) as well as an interaction term between the factors (e.g. emotional eating x physical activity). Statistically significant interaction effects were probed using simple slope analysis.

#### Associations between BMI trajectories and mental health outcomes

In a second series of regression models, we tested whether BMI trajectories were associated with well-being, quality of life, general self-rated health, and body satisfaction at follow-up (2024), adjusting for the covariates noted above as well as anxiety and depressive symptoms at baseline. To test whether sex moderated the association between BMI trajectories and body satisfaction, we included an interaction term between BMI trajectories and sex.

#### Factors contributing to the association between BMI trajectories and mental health

Structural equation modelling with maximum likelihood estimation was used to examine: (1) the extent to which emotional eating mediated the association between depressive and anxiety symptoms at baseline and BMI trajectories (individual BMI slopes as continuous outcomes), (2) the extent to which depressive and anxiety symptoms and emotional eating mediated the association between financial hardship and BMI trajectories, and (3) the extent to which body satisfaction and self-rated health mediated the association between BMI trajectories and mental health outcomes at follow-up (one model per outcome) after adjustment for covariates. A mediating effect was the presence of a significant indirect effect (the product of the direct paths) [35]. The Monte Carlo method (5000 samples) was used to estimate standardised indirect effects with 95% confidence intervals (CIs) [36]. Indirect effects were interpreted as: 0.003 (0.05 × 0.05) is a small but meaningful indirect effect, 0.01 (0.10 × 0.10) is a moderate indirect effect, and 0.06 (0.25 × 0.25) is a large indirect effect [37]. Model fit was assessed using the root mean square error of approximation (RMSEA), comparative fit index (CFI), and Tucker-Lewis index (TLI) [38].

We ran the following sensitivity analyses: (1) excluding individuals who were underweight at any time (*N* = 288), (2) excluding individuals who had weight change ≥ 30 kg per year, which could reflect an underlying health condition (*N* = 3), and (3) excluding individuals who were pregnant during the study (*N* = 196). Statistical analyses were conducted using Stata® version 16 (Stata Corporation, College Station, TX, USA).

## Results

### Descriptive statistics

Table 1 shows characteristics of the study sample. Participants were on average 51 years old (SD 14; range 18–97 years), 59% were women, 65% were educated to tertiary level, 23% had comorbidities, and the mean BMI at inclusion was 24.5 kg/m^2^ (SD 4.2). Compared to those with data at inclusion (*N* = 13,260), participants were older (mean age 51 years versus 47 years, *p* < 0.001), slightly more were educated to tertiary level (65% versus 64%, *p* < 0.001), and slightly more had comorbidities (23% versus 21%, *p* < 0.001). Depressive symptom scores were slightly lower among participants than the total sample with data at inclusion (mean score 0.9 versus 1), but there was no difference in anxiety scores or BMI at inclusion between groups (see supplementary Table 2).

**Table 1:** Characteristics of the total study sample, and by sex (statistics are mean (SD) or % (*n*)).

	Total	Men	Women	*p*-value
	*N* = 7388	*N* = 2987	*N* = 4401	
Age at inclusion, years	50.7 (13.6)	52.5 (13.6)	49.5 (13.5)	<0.001
Education level				0.57
Tertiary	65.2 (4806)	65.8 (1963)	64.7 (2843)	
Secondary	31.1 (2292)	30.4 (906)	31.6 (1386)	
Primary	3.8 (277)	3.8 (113)	3.7 (164)	
Physical health condition				0.64
No	77.2 (5703)	77.5 (2314)	77.0 (3389)	
Yes	22.8 (1685)	22.5 (673)	23.0 (1012)	
Frequency of moderate physical activity, 1 = never, 6 = every day	4.0 (1.5)	4.0 (1.5)	4.0 (1.5)	0.28
Frequency of vigorous physical activity, 1 = never, 6 = every day	2.7 (1.4)	2.9 (1.4)	2.6 (1.3)	<0.001
Daily portions of fruit and vegetables	3.1 (1.8)	2.8 (1.7)	3.3 (1.8)	<0.001
Binge drinking				<0.001
Less than once a month	67.1 (4226)	55.9 (1427)	74.8 (2799)	
Once a month or more	32.9 (2070)	44.1 (1127)	25.2 (943)	
Sleep duration per night				<0.001
≥ 7 h	69.1 (5094)	65.2 (1942)	71.7 (3152)	
< 7 h	30.9 (2279)	34.8 (1036)	28.3 (1243)	
Anti-depressant drug use				<0.001
No	96.3 (7115)	97.6 (2915)	95.4 (4200)	
Yes	3.7 (273)	2.4 (72)	4.6 (201)	
Emotional eating score, range 1–5	2.4 (1.2)	2.1 (1.1)	2.6 (1.2)	<0.001
Social support score, range 3–14	10.3 (1.9)	10.3 (1.9)	10.3 (1.9)	0.32
Financial difficulties				0.004
No difficulties	56.7 (4190)	58.8 (1756)	55.3 (2434)	
Average difficulties	30.2 (2232)	29.3 (874)	30.9 (1358)	
Important difficulties	6.6 (485)	6.4 (192)	6.7 (293)	
I don’t wish to respond	6.5 (477)	5.5 (163)	7.1 (314)	
Self-esteem score, range 1–5	4.2 (0.7)	4.2 (0.7)	4.1 (0.7)	<0.001
Self-efficacy score, range 1–5	4.0 (0.6)	4.1 (0.6)	4.0 (0.6)	<0.001
Life mastery score, range 4–16	12.7 (2.9)	13.1 (3.0)	12.5 (2.9)	<0.001
Loneliness score, range 3–9	4.6 (1.7)	4.3 (1.6)	4.7 (1.7)	<0.001
Depressive symptoms, range 0–6	0.9 (1.2)	0.8 (1.2)	1.0 (1.2)	<0.001
Anxiety symptoms, range 0–6	1.2 (1.3)	1.0 (1.3)	1.3 (1.3)	<0.001
BMI	24.5 (4.2)	25.5 (3.8)	23.9 (4.4)	<0.001
BMI category				<0.001
Healthy weight	58.5 (4322)	49.3 (1473)	64.8 (2849)	
Underweight	2.8 (210)	0.8 (24)	4.2 (186)	
Overweight	28.6 (2116)	39.1 (1168)	21.5 (948)	
Obesity	9.4 (697)	10.2 (306)	8.9 (391)	
Severe obesity	0.6 (41)	0.5 (15)	0.6 (26)	

**Fig. 1:**
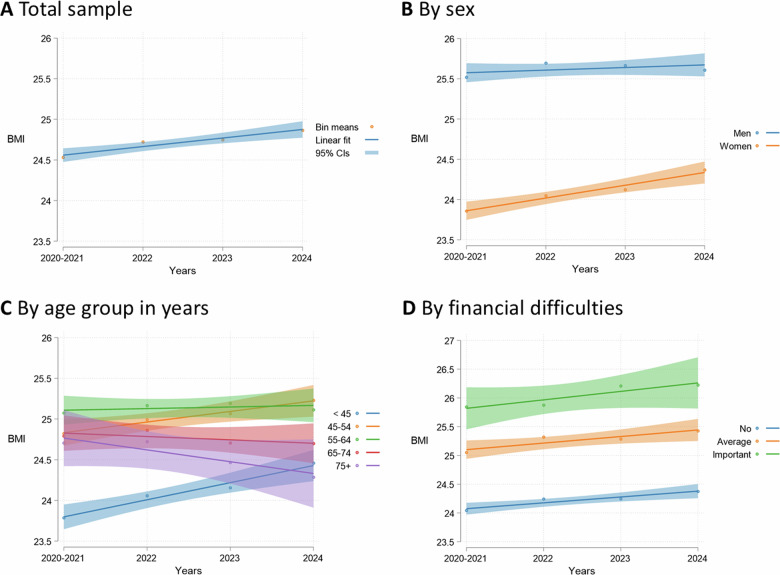
Trends in BMI over time for the total study sample, and by sex, age group, and financial difficulties (*N* = 7388). Results are means and 95% confidence intervals.

**Fig. 2:**
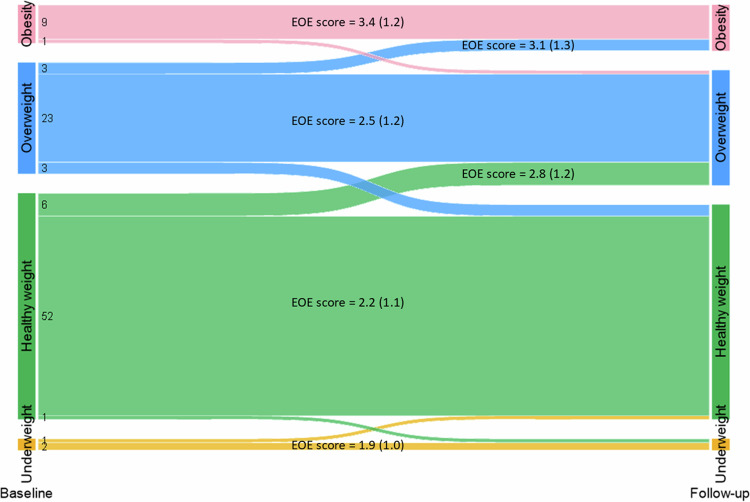
BMI category transitions from baseline (inclusion) to latest follow-up (*N* = 7388). Values to the left are the rounded percent within each group. EOE Score = mean emotional eating score and standard deviation in parentheses. For ease of presentation, those with stable obesity (*N* = 580), stable severe obesity (*N* = 30), and those who transitioned between obesity and severe obesity (*N* = 24) are combined in one group.

On average, there was a slight increase in BMI over time in the study sample (0.4 kg/m^2^), with mean BMI falling at the upper end of the healthy weight category by 2024 (see Fig. 1). The increase was most notable for women and younger age groups (< 55 years), and BMI level was consistently higher among those with financial difficulties (see Fig. 1). Most of the sample (86%) had the same BMI classification at follow-up as at baseline (see Fig. 2). 8% of the sample had an upward BMI classification trajectory, transitioning from healthy weight to overweight (5%), from overweight to obesity, or from obesity to severe obesity (3%). 5% had a downward BMI classification trajectory. By 2024, 43% of the sample had overweight or obesity.

### Behavioural and psychosocial factors associated with BMI trajectories

**Fig. 3:**
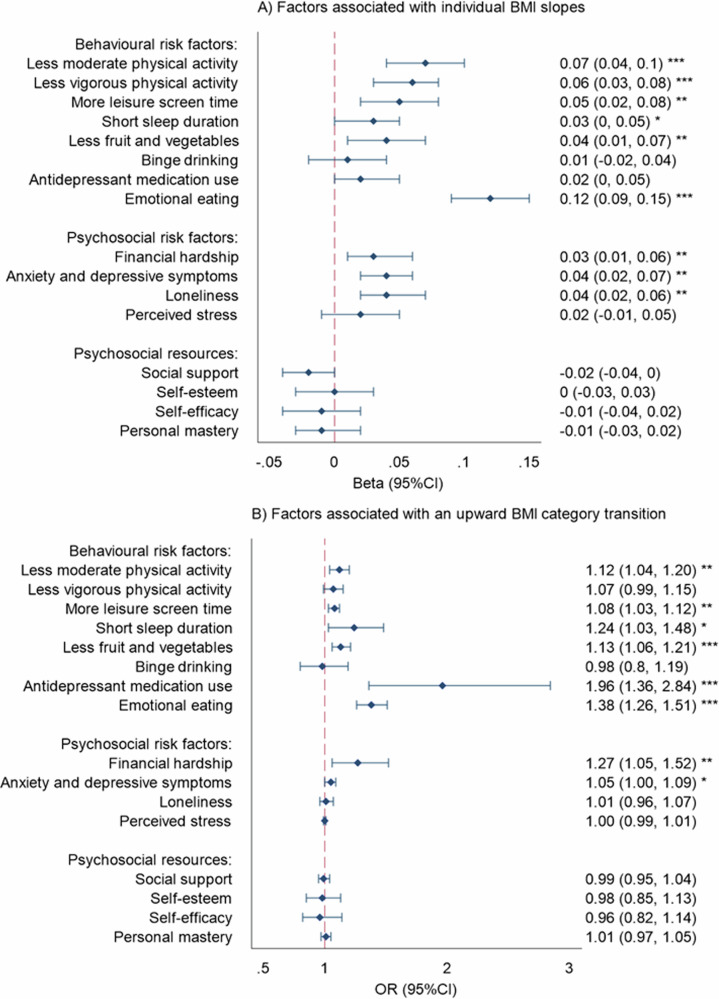
Behavioural and psychosocial factors associated with BMI trajectories. Models adjusted for age, sex, education, and physical health condition. Results are standardised betas **A** and ORs **B** with 95% confidence intervals. *p < 0.05, **p < 0.01, ***p < 0.001. Short sleep duration = < 7 h. Reference categories for categorical variables are as follows: No short sleep duration, no binge drinking, no anti-depressant medication use, and no financial hardship. Total *N* per model: physical activity (*N* = 5267), screen time (*N* = 4017), sleep duration (*N* = 7358), fruit and vegetable consumption (*N* = 4313), binge drinking (*N* = 4579), anti-depressant medication use (*N* = 7373), emotional eating (*N* = 4313), financial hardship (*N* = 7370), anxiety and depressive symptoms (*N* = 5657), loneliness (*N* = 7283), perceived stress (*N* = 5179), social support (*N* = 7233), self-esteem (*N* = 4313), self-efficacy (*N* = 4313), personal mastery (*N* = 5872).

Figure 3 shows the factors associated with BMI trajectories. Behavioural factors associated with increasing BMI included less moderate and vigorous physical activity, less fruit and vegetable consumption, more leisure screen time, short sleep duration, antidepressant medication use, and emotional eating. Psychosocial factors associated with increasing BMI were financial hardship, loneliness, and depressive and anxiety symptoms. Examination of significant interaction effects showed that the association between emotional eating and BMI trajectories was stronger among women (marginal effect = 0.04 [0.03, 0.05], *p* < 0.001) than men (marginal effect = 0.02 [0.00, 0.03], *p* = 0.014); and stronger among individuals who never engaged in moderate or vigorous physical activity (marginal effect = 0.05 [0.03, 0.07], *p* < 0.001) than those who engaged in moderate or vigorous physical activity most days (marginal effect = 0.02 [0.00, 0.03], *p* = 0.038). There were no other significant interaction effects. Associations between the behavioural and psychosocial factors with BMI levels are included in Supplementary Fig. 1.

### Associations between BMI trajectories and mental health outcomes

**Fig. 4:**
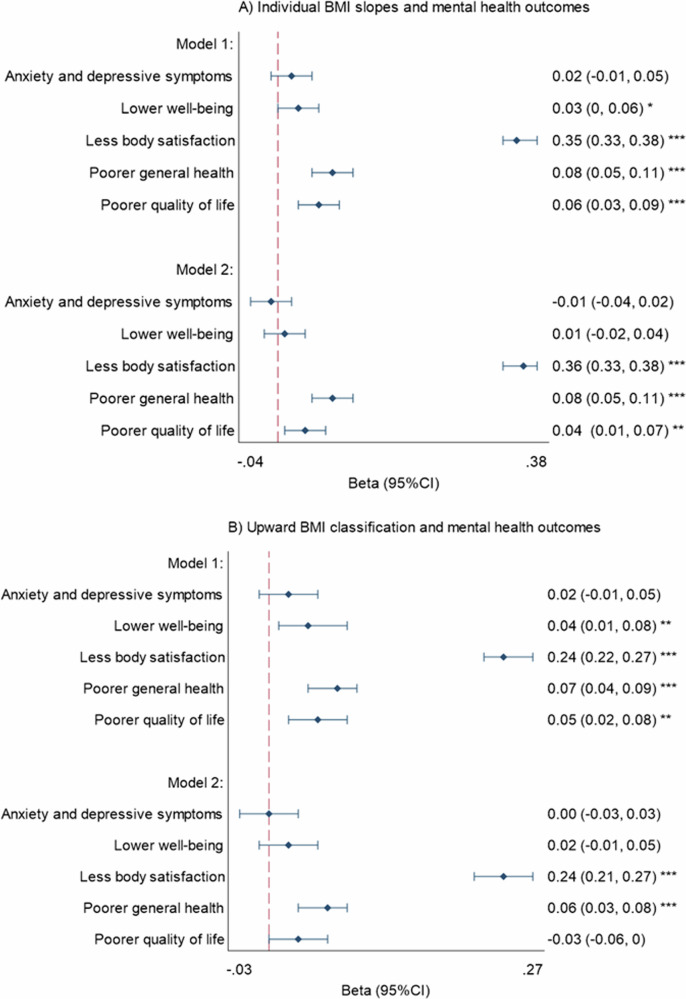
Associations between BMI trajectories and mental health outcomes at follow-up (2024). Models adjusted for age, sex, education, physical health condition (model 1), and anxiety and depressive symptoms at baseline (model 2). Results are standardised betas with 95% confidence intervals. *p < 0.05, **p < 0.01, ***p < 0.001. *N* = 4313.

Figure 4 shows the associations between BMI trajectories and mental health outcomes at follow-up (2024). Increasing BMI was negatively associated with body satisfaction and quality of life, but not anxiety and depressive symptoms, after adjustment for age, sex, education, physical comorbidities, and anxiety and depressive symptoms at baseline. Increasing BMI was also associated with poorer self-rated health at follow-up, after adjustment for self-rated health at baseline. Examination of significant interaction effects showed that the association between BMI trajectories and body dissatisfaction was stronger among women (marginal effect = −2.91 [−3.19, −2.63], *p* < 0.001) than men (marginal effect = −2.33 [−2.72, −1.94], *p* < 0.001). Associations between BMI levels and mental health outcomes are included in Supplementary Fig. 2.

### Factors contributing to the association between BMI trajectories and mental health

Structural equation modelling showed there was a significant large indirect effect (β = 0.021; 95% CI, 0.014–0.028; *p* < 0.001) indicating that emotional eating partly mediated the association between anxiety and depressive symptoms at baseline and BMI trajectories. Depressive and anxiety symptoms and emotional eating also partly mediated the association between financial hardship and BMI trajectories: there was a small indirect effect via anxiety and depressive symptoms (β = 0.005; 95% CI, 0.002–0.009; *p* = 0.003), and a small indirect effect via emotional eating (β = 0.007; 95% CI, 0.003–0.011; *p* = 0.001).

Both self-rated health and body satisfaction mediated the association between BMI trajectories and quality of life at follow-up after adjustment for anxiety and depressive symptoms at baseline: there was a moderate indirect effect of BMI trajectories on quality of life via self-rated health (β = −0.031; 95% CI, −0.045–-0.018; *p* < 0.001), and a large indirect effect of BMI trajectories on quality of life via body dissatisfaction (β = −0.078; 95% CI, −0.091–-0.066; *p* < 0.001). All tested models had acceptable fit (RMSEA ≤ 0.06, CFI ≥ 0.95, and TLI ≥ 0.95). All results were the same when running the sensitivity analyses.

## Discussion

### Summary of results

In this adult population-based cohort, we tested hypothesised pathways linking BMI trajectories and mental health. Factors associated with increasing BMI over 4-year follow-up included financial hardship, short sleep duration, less physical activity, more leisure screen time, depressive and anxiety symptoms, and emotional eating. Associations between emotional eating and BMI trajectories were strongest among women and those who were less physically active. Increasing BMI was associated with body dissatisfaction and poorer quality of life at 4-year follow-up after adjustment for anxiety and depressive symptoms at baseline. Emotional eating partly explained the association between anxiety and depressive symptoms at baseline and BMI trajectories, as well as the association between financial hardship and BMI trajectories. Body dissatisfaction and poorer self-rated health partly explained the association between BMI trajectories and quality of life at follow-up after adjustment for anxiety and depressive symptoms at baseline.

### Behavioural and psychosocial factors associated with BMI trajectories

Emotional eating was consistently associated with increasing BMI, whether measured continuously as individual slopes or as an upward BMI category transition. Previous research has shown that emotional eating is associated with weight outcomes, including weight gain over time [39]. Our findings corroborate and extend this research in a population-based sample, with BMI assessment at multiple time points, and assessment of moderating and mediating pathways. In line with previous research among adult employees [40], the association between emotional eating and BMI trajectories was stronger than that for other behavioural factors such as physical activity and fruit and vegetable consumption. This and our study did not have direct measures of overall diet quality and caloric intake, but emotional eating is characterised by the excessive consumption of hyperpalatable energy-dense foods [41, 42]. It is therefore not surprising that fruit and vegetable consumption had smaller individual effects on BMI trajectories than emotional eating did. There is general agreement that multiple interacting factors contribute to weight-related outcomes, and overall diet quality alongside physical activity plays an important role. Consistent with previous cross-sectional research [14], we found that the association between emotional eating and increasing BMI was stronger among individuals who were less physically active. Emotional eaters who engage in little physical activity represent a group that is particularly susceptible to weight gain and may benefit from weight management strategies targeting both eating style and activity level. We did not replicate the previous finding that emotional eaters with shorter sleep duration are particularly susceptible to weight gain [12], and we suggest that further research examines the interplay between emotional eating and sleep using objective measures in a clinical setting.

To our knowledge, only a few studies have assessed the moderating effect of psychological traits or resources in the relationship between emotional eating and weight change [15]. Previous research focused on other control-related constructs (namely impulsivity and consideration of future consequences) and found that while there was a moderating effect on weight status, there was no moderating effect on weight change [15]. This and our finding could be due to a lack of statistical power in the detection of interaction effects [43], or because the duration of follow-up was too short to observe a meaningful difference. Alternatively, eating-self-regulatory capacity may be more closely related to emotional eating and BMI change than general life mastery, acting as either a moderator or mediator of their association [44]. Indeed, recent research suggests that fostering self-regulatory skills can mitigate unhealthy eating behaviours associated with the tendency for emotional eating [45]. Further research into their role in weight gain is needed as public health strategies aimed at enhancing self-regulatory skills might be effective in reducing the risk of metabolic diseases for susceptible individuals exposed to unhealthful food environments.

### BMI trajectories associated with mental health outcomes

The association between excess weight and poor mental health, especially depression, is well established [6]. Our study is one of few to examine associations between weight change and mental health outcomes. Consistent with recent research [10], we found that increasing BMI was associated with poorer mental health at follow-up. The association was apparent for a range of mental health outcomes, including positive psychology constructs. After adjustment for prior mental health, associations were attenuated but held for quality of life and body satisfaction. Although anxiety and depressive symptoms at baseline were associated with BMI trajectories, BMI trajectories were not associated with anxiety and depressive symptoms at follow-up. As there are few studies examining the association between weight change and mental health over time, it would be premature to conclude that BMI trajectories are not associated with subsequent anxiety and depressive symptoms. Indeed, a recent study found that weight gain from childhood to mid-adulthood was associated with a higher risk of depression in adulthood [10]. Associations between BMI trajectories and anxiety and depression may therefore be evident over longer follow-up, and when using clinical measures of mental health, which are more precise than self-report questionnaires [5]. Previous research has shown that the association between obesity and depression is stronger than the association between overweight and depression, reflecting a dose-response gradient [5]. The same could be true for weight change i.e. the greater the weight gain, the greater the risk of subsequent depression.

### Factors contributing to the association between BMI trajectories and mental health

Emotional eating partly mediated the effect of anxiety and depressive symptoms on increasing BMI over time, consistent with previous research examining BMI change across two time points [12]. This finding is in line with the notion that individuals who are anxious or depressed are more likely to be emotional eaters, which can increase their risk for weight gain. Emotional eating may be an attempt to cope with negative emotions, and/or emotional eaters may confuse bodily states related to emotions with physiological internal states guiding satiety and hunger [46]. Appetitive traits tend to be inter-related [24], therefore, emotional eaters may engage in other eating behaviours that increase their risk for weight gain. Anxiety and depressive symptoms and emotional eating also mediated the association between financial difficulties and BMI trajectories, extending previous cross-sectional research [13]. This finding further supports the theory that psychological distress and maladaptive coping contribute to the association between social disadvantage and obesity [47]. Moreover, socially disadvantaged groups have greater exposure to obesogenic environments than their more advantaged counterparts, rendering them more likely to engage in maladaptive eating behaviours [48, 49].

Body dissatisfaction mediated the association between BMI trajectories and quality of life at follow-up, corroborating and extending recent longitudinal research in adolescents [16], and cross-sectional research in women [50]. Moreover, the association between BMI trajectories and body dissatisfaction was stronger for women than men, which could be explained by sex-specific societal beauty ideals, which place greater value on thinness for women and on muscular bodies for men [51]. Poorer self-rated health also partly mediated the association between BMI trajectories and quality of life, which is in line with the hypothesis that obesity and weight gain increase the risk of poor mental health via both health and appearance concern pathways [52].

### Practical implications

Targeting modifiable behaviours such as physical activity, sleep, diet, and eating style may simultaneously improve BMI and mental health via anti-inflammatory pathways that interact with gut microbiota [11, 53]. Randomised controlled trials of adult behavioural weight loss interventions show improvements in depression, quality of life, and self-efficacy at intervention-end and 12-month follow-up [54]. However, there is insufficient evidence to assess the impact on anxiety, emotional eating, body image, self-esteem, and stress. Further high-quality trials measuring a range of mental health outcomes over longer durations are needed to determine the impact of intentional weight loss on mental health. Weight loss strategies should avoid weight stigma, which contributes to the association between excess weight and poor mental health [55]. Interventions addressing body dissatisfaction show encouraging results in women [56], but more trials are needed. Efforts to reduce weight stigma in society could help to reduce the psychological burden of overweight and obesity.

### Strengths and limitations

Strengths include the population-based longitudinal design, consideration of a range of behavioural and psychosocial factors in relation to BMI trajectories, as well as hypothesised interactions between factors, and the assessment of mediating pathways linking BMI trajectories and mental health. Limitations are the use of self-reported height and weight, although the results of this and other studies support their validity. While BMI is often used as a measure of body adiposity, it does not distinguish between excess fat, muscle, or bone mass. We could not distinguish between typical and atypical depression, which may be differently associated with body adiposity [57], nor did we have clinical measures of mental health, which are more strongly associated with weight [5]. We measured emotional eating, but other eating behaviours also play a role in BMI trajectories [24]. We assessed emotional eating and fruit and vegetable consumption once at the end of the study period. Research indicates that eating behaviour traits such as emotional eating are largely stable during childhood [58, 59] and into adulthood [60]; therefore, we would expect little change in emotional eating during the study period. However, we cannot exclude the possibility that eating behaviours changed following weight change, such as weight gain leading to an increase in emotional eating [61]. Studies with repeated measures of eating behaviours and BMI are needed to provide further insights. For example, some research in children suggests that the relationship between emotional eating and BMI is bidirectional [62]. We did not test all possible interaction effects or mediating pathways, and we acknowledge that establishing true causality is difficult and beyond the scope of our analyses.

## Conclusions

Understanding the role of modifiable risk factors for overweight and obesity, as well as associated health outcomes, is central to developing effective prevention and intervention strategies. Our study highlights emotional eating and body dissatisfaction as important factors associated with BMI trajectories and mental health, and to be considered in weight management and mental health promotion strategies.

## Supplementary information

Supplemental information

## Data Availability

The data are available on request from the corresponding author.

## References

[1] Phelps NH, Singleton RK, Zhou B, Heap RA, Mishra A, Bennett JE, et al. Worldwide trends in underweight and obesity from 1990 to 2022: a pooled analysis of 3663 population-representative studies with 222 million children, adolescents, and adults. Lancet. 2024;403:1027–50. 38432237 10.1016/S0140-6736(23)02750-2 PMC7615769

[2] NCD Risk Factor Collaboration. Trends in adult body-mass index in 200 countries from 1975 to 2014: a pooled analysis of 1698 population-based measurement studies with 19·2 million participants. Lancet. 2016;387:1377–96. 27115820 10.1016/S0140-6736(16)30054-X PMC7615134

[3] Angelantonio ED, Bhupathiraju SN, Wormser D, Gao P, Kaptoge S, Gonzalez AB, et al. Body-mass index and all-cause mortality: individual-participant-data meta-analysis of 239 prospective studies in four continents. Lancet. 2016;388:776–86. 27423262 10.1016/S0140-6736(16)30175-1 PMC4995441

[4] Okunogbe A, Nugent R, Spencer G, Ralston J, Wilding J. Economic impacts of overweight and obesity: current and future estimates for eight countries. BMJ Glob Health. 2021;6:e006351. 34737167 10.1136/bmjgh-2021-006351 PMC8487190

[5] Luppino FS, de Wit LM, Bouvy PF, Stijnen T, Cuijpers P, Penninx BWJH, et al. Overweight, obesity, and depression: a systematic review and meta-analysis of longitudinal studies. Arch Gen Psychiatry. 2010;67:220–9. 20194822 10.1001/archgenpsychiatry.2010.2

[6] Steptoe A, Frank P. Obesity and psychological distress. Philos Trans R Soc B. 2023;378:20220225. 10.1098/rstb.2022.0225 PMC10475872 37661745

[7] Frank P, Jokela M, Batty GD, Lassale C, Steptoe A, Kivimäki M. Overweight, obesity, and individual symptoms of depression: a multicohort study with replication in UK Biobank. Brain Behav Immun. 2022;105:192–200. 35853559 10.1016/j.bbi.2022.07.009 PMC10499756

[8] Frank P, Batty GD, Pentti J, Jokela M, Poole L, Ervasti J, et al. Association between depression and physical conditions requiring hospitalization. JAMA Psychiatry. 2023;80:690–9. 37133850 10.1001/jamapsychiatry.2023.0777 PMC10157511

[9] Milaneschi Y, Simmons WK, van Rossum EFC, Penninx BW. Depression and obesity: evidence of shared biological mechanisms. Mol Psychiatry. 2019;24:18–33. 29453413 10.1038/s41380-018-0017-5

[10] Gallagher C, Pirkis J, Lambert KA, Perret JL, Ali GB, Lodge CJ, et al. Life course BMI trajectories from childhood to mid-adulthood are differentially associated with anxiety and depression outcomes in middle age. Int J Obes. 2023;47:661–8. 10.1038/s41366-023-01312-6 PMC10359183 37161067

[11] Yu T, Chen C, Yang Y, Wang M, Yang Y, Feng W, et al. Dissecting the association between gut microbiota, body mass index and specific depressive symptoms: a mediation Mendelian randomisation study. Gen Psychiatry. 2024;37:e101412. 10.1136/gpsych-2023-101412 PMC11227829 38975363

[12] Konttinen H, van Strien T, Männistö S, Jousilahti P, Haukkala A. Depression, emotional eating and long-term weight changes: a population-based prospective study. Int J Behav Nutr Phys Act. 2019;16:28. 30894189 10.1186/s12966-019-0791-8 PMC6427874

[13] Spinosa J, Christiansen P, Dickson JM, Lorenzetti V, Hardman CA. From socioeconomic disadvantage to obesity: the mediating role of psychological distress and emotional eating. Obesity. 2019;27:559–64. 30821100 10.1002/oby.22402 PMC6593860

[14] Dohle S, Hartmann C, Keller C. Physical activity as a moderator of the association between emotional eating and BMI: evidence from the Swiss Food Panel. Psychol Health. 2014;29:1062–80. 24689843 10.1080/08870446.2014.909042

[15] Bénard M, Bellisle F, Etilé F, Reach G, Kesse-Guyot E, Hercberg S, et al. Impulsivity and consideration of future consequences as moderators of the association between emotional eating and body weight status. Int J Behav Nutr Phys Act. 2018;15:84. 30189878 10.1186/s12966-018-0721-1 PMC6127957

[16] Blundell E, Stavola BLD, Kellock MD, Kelly Y, Lewis G, McMunn A, et al. Longitudinal pathways between childhood BMI, body dissatisfaction, and adolescent depression: an observational study using the UK Millennium Cohort Study. Lancet Psychiatry. 2024;11:47–55. 38101872 10.1016/S2215-0366(23)00365-6 PMC11139652

[17] Baysson H, Pennacchio F, Wisniak A, Zaballa M-E, Pullen N, Collombet P, et al. The Specchio-COVID19 cohort study: a longitudinal follow-up of SARS-CoV-2 serosurvey participants in the canton of Geneva, Switzerland. BMJ Open. 2022;12:e055515. 35105645 10.1136/bmjopen-2021-055515 PMC8804307

[18] de Mestral C, Stringhini S, Guessous I, Jornayvaz FR. Thirteen-year trends in the prevalence of diabetes in an urban region of Switzerland: a population-based study. Diabet Med. 2020;37:1374–8. 31814147 10.1111/dme.14206

[19] Stringhini S, Wisniak A, Piumatti G, Azman AS, Lauer SA, Baysson H, et al. Seroprevalence of anti-SARS-CoV-2 IgG antibodies in Geneva, Switzerland (SEROCoV-POP): a population-based study. Lancet. 2020;396:313–9. 32534626 10.1016/S0140-6736(20)31304-0 PMC7289564

[20] Stringhini S, Zaballa M-E, Perez-Saez J, Pullen N, de Mestral C, Picazio A, et al. Seroprevalence of anti-SARS-CoV-2 antibodies after the second pandemic peak. Lancet Infect Dis. 2021;21:600–1. 33539733 10.1016/S1473-3099(21)00054-2 PMC8063076

[21] Stringhini S, Zaballa M-E, Pullen N, de Mestral C, Perez-Saez J, Dumont R, et al. Large variation in anti-SARS-CoV-2 antibody prevalence among essential workers in Geneva, Switzerland. Nat Commun. 2021;12:3455. 34103517 10.1038/s41467-021-23796-4 PMC8187639

[22] Martínez-González MA, García-Arellano A, Toledo E, Salas-Salvadó J, Buil-Cosiales P, Corella D, et al. A 14-Item mediterranean diet assessment tool and obesity indexes among high-risk subjects: the PREDIMED trial. PLoS ONE. 2012;7:e43134. 22905215 10.1371/journal.pone.0043134 PMC3419206

[23] Hirshkowitz M, Whiton K, Albert SM, Alessi C, Bruni O, DonCarlos L, et al. National Sleep Foundation’s updated sleep duration recommendations: final report. Sleep Health. 2015;1:233–43. 29073398 10.1016/j.sleh.2015.10.004

[24] Hunot C, Fildes A, Croker H, Llewellyn CH, Wardle J, Beeken RJ. Appetitive traits and relationships with BMI in adults: development of the adult eating behaviour questionnaire. Appetite. 2016;105:356–63. 27215837 10.1016/j.appet.2016.05.024 PMC4990060

[25] Petrovic D, Carmeli C, Sandoval JL, Bodinier B, Chadeau-Hyam M, Schrempft S, et al. Life-course socioeconomic factors are associated with markers of epigenetic aging in a population-based study. Psychoneuroendocrinology. 2023;147:105976. 36417838 10.1016/j.psyneuen.2022.105976

[26] Monteiro RP, Coelho GL, de H, Hanel PHP, de Medeiros ED, da Silva PDG. The efficient assessment of self-esteem: proposing the brief rosenberg self-esteem scale. Appl Res Qual Life. 2022;17:931–47.

[27] Schuler D, Tuch A, Peter C La santé psychique en Suisse. Monitorage 2020 (Obsan Rapport 15/2020). 2020.

[28] Pearlin LI, Menaghan EG, Lieberman MA, Mullan JT. The Stress Process. J Health Soc Behav. 1981;22:337–56. 7320473

[29] Décieux JP, Sischka PE, Schumacher A, Willems H. Psychometrical properties of a French version of the general self-efficacy short scale (ASKU). Swiss J Psychol. 2020;79:15–25.

[30] Hughes ME, Waite LJ, Hawkley LC, Cacioppo JT. A short scale for measuring loneliness in large surveys: results from two population-based studies. Res Aging. 2004;26:655–72. 18504506 10.1177/0164027504268574 PMC2394670

[31] Kroenke K, Spitzer RL, Williams JBW, Löwe B. An ultra-brief screening scale for anxiety and depression: the PHQ–4. Psychosomatics. 2009;50:613–21. 19996233 10.1176/appi.psy.50.6.613

[32] Cohen S Perceived stress in a probability sample of the United States. In: Spacapan S & Oskamp S (eds). *The Social Psychology of Health*. Sage Publications, Inc: Thousand Oaks, CA, US, 1988. pp 31–67.

[33] Bardi L, Arnaud C, Bagès C, Langlois F, Rousseau A. Translation and validation of a state-measure of body image satisfaction: the body image state scale. Front Psychol. 2021;12:724710. 34777102 10.3389/fpsyg.2021.724710 PMC8581347

[34] Topp CW, Østergaard SD, Søndergaard S, Bech P. The WHO-5 Well-Being Index: A systematic review of the literature. Psychother Psychosom. 2015;84:167–76. 25831962 10.1159/000376585

[35] Zhao X, Lynch JG Jr, Chen Q. Reconsidering Baron and Kenny: myths and truths about mediation analysis. J Consum Res. 2010;37:197–206.

[36] Mehmetoglu M medsem: a Stata package for statistical mediation analysis. *medsem: a Stata package for statistical mediation analysis* 2018. 10.1504/IJCEE.2018.10007883.

[37] Keith TZ *Multiple Regression and Beyond: An Introduction to Multiple Regression and Structural Equation Modeling*. Routledge, 2014.

[38] Hu L, Bentler PM. Cutoff criteria for fit indexes in covariance structure analysis: conventional criteria versus new alternatives. Struct Equ Model. 1999;6:1–55.

[39] Frayn M, Knäuper B. Emotional eating and weight in adults: a review. Curr Psychol. 2018;37:924–33.

[40] Koenders PG, van Strien T. Emotional eating, rather than lifestyle behavior, drives weight gain in a prospective study in 1562 employees. J Occup Environ Med. 2011;53:1287. 22027541 10.1097/JOM.0b013e31823078a2

[41] Konttinen H, Männistö S, Sarlio-Lähteenkorva S, Silventoinen K, Haukkala A. Emotional eating, depressive symptoms and self-reported food consumption. A population-based study. Appetite. 2010;54:473–9. 20138944 10.1016/j.appet.2010.01.014

[42] Fuente González CE, Chávez-Servín JL, de la Torre-Carbot K, Ronquillo González D, Aguilera Barreiro M, de los Á, et al. Relationship between emotional eating, consumption of hyperpalatable energy-dense foods, and indicators of nutritional status: a systematic review. J Obes. 2022;2022:4243868. 35634585 10.1155/2022/4243868 PMC9132695

[43] Rothman KJ, Greenland S, Lash T, Modern epidemiology. Philadelphia: Wolters Kluwer Health/Lippincott Williams & Wilkins; 2008 Sep 20.

[44] Ling J, Zahry NR. Relationships among perceived stress, emotional eating, and dietary intake in college students: eating self-regulation as a mediator. Appetite. 2021;163:105215. 33774134 10.1016/j.appet.2021.105215

[45] Annesi JJ, Johnson PH. Mitigation of the effects of emotional eating on sweets consumption by treatment-associated self-regulatory skills usage in emerging adult and middle-age women with obesity. Appetite. 2020;155:104818. 32750395 10.1016/j.appet.2020.104818

[46] Konttinen H. Emotional eating and obesity in adults: the role of depression, sleep and genes. Proc Nutr Soc. 2020;79:283–9. 32213213 10.1017/S0029665120000166

[47] Hemmingsson E. A new model of the role of psychological and emotional distress in promoting obesity: conceptual review with implications for treatment and prevention. Obes Rev. 2014;15:769–79. 24931366 10.1111/obr.12197

[48] Schrempft S, van Jaarsveld CHM, Fisher A, Herle M, Smith AD, Fildes A, et al. Variation in the heritability of child body mass index by obesogenic home environment. JAMA Pediatr. 2018;172:1153–60. 30285028 10.1001/jamapediatrics.2018.1508 PMC6396810

[49] Llewellyn C, Wardle J. Behavioral susceptibility to obesity: gene–environment interplay in the development of weight. Physiol Behav. 2015;152:494–501. 26166156 10.1016/j.physbeh.2015.07.006

[50] Grano C, Vacca M, Lombardo C. The relationship between body mass index, body dissatisfaction and mood symptoms in pregnant women. J Clin Med. 2024;13:2424. 38673697 10.3390/jcm13082424 PMC11051092

[51] Murray SB, Griffiths S, Mond JM. Evolving eating disorder psychopathology: conceptualising muscularity-oriented disordered eating. Br J Psychiatry. 2016;208:414–5. 27143005 10.1192/bjp.bp.115.168427

[52] Markowitz S, Friedman MA, Arent SM. Understanding the relation between obesity and depression: causal mechanisms and implications for treatment. Clin Psychol Sci Pr. 2008;15:1–20.

[53] Chu K, Cadar D, Iob E, Frank P. Excess body weight and specific types of depressive symptoms: is there a mediating role of systemic low-grade inflammation? Brain Behav Immun. 2023;108:233–44. 36462595 10.1016/j.bbi.2022.11.016 PMC10567582

[54] Jones RA, Lawlor ER, Birch JM, Patel MI, Werneck AO, Hoare E, et al. The impact of adult behavioural weight management interventions on mental health: a systematic review and meta-analysis. Obes Rev. 2021;22:e13150. 33103340 10.1111/obr.13150 PMC7116866

[55] Jackson SE, Beeken RJ, Wardle J. Obesity, perceived weight discrimination, and psychological well-being in older adults in England. Obesity. 2015;23:1105–11. 25809860 10.1002/oby.21052 PMC4414736

[56] Guest E, Costa B, Williamson H, Meyrick J, Halliwell E, Harcourt D. The effectiveness of interventions aiming to promote positive body image in adults: a systematic review. Body Image. 2019;30:10–25. 31077956 10.1016/j.bodyim.2019.04.002

[57] Lamers F, Milaneschi Y, de Jonge P, Giltay EJ, Penninx BWJH. Metabolic and inflammatory markers: associations with individual depressive symptoms. Psychol Med. 2018;48:1102–10. 28889804 10.1017/S0033291717002483

[58] Ashcroft J, Semmler C, Carnell S, van Jaarsveld CHM, Wardle J. Continuity and stability of eating behaviour traits in children. Eur J Clin Nutr. 2008;62:985–90. 17684526 10.1038/sj.ejcn.1602855

[59] Jansen E, Thapaliya G, Beauchemin J, D’Sa V, Deoni S, Carnell S. The development of appetite: tracking and age-related differences in appetitive traits in childhood. Nutrients. 2023;15:1377. 36986108 10.3390/nu15061377 PMC10056659

[60] Dubois L, Bédard B, Goulet D, Prud’homme D, Tremblay RE, Boivin M. Eating behaviors, dietary patterns and weight status in emerging adulthood and longitudinal associations with eating behaviors in early childhood. Int J Behav Nutr Phys Act. 2022;19:139. 36384744 10.1186/s12966-022-01376-z PMC9670577

[61] MacLean PS, Blundell JE, Mennella JA, Batterham RL. Biological control of appetite: a daunting complexity. Obesity. 2017;25:S8–S16. 28229538 10.1002/oby.21771 PMC5407690

[62] Derks IPM, Sijbrands EJG, Wake M, Qureshi F, van der Ende J, Hillegers MHJ, et al. Eating behavior and body composition across childhood: a prospective cohort study. Int J Behav Nutr Phys Act. 2018;15:96. 30285789 10.1186/s12966-018-0725-x PMC6167809

