# Supplementary material for: Pathways linking BMI trajectories and mental health in an adult population-based cohort: role of emotional eating and body dissatisfaction
Source: Int J Obes (Lond). 2025 Apr 7;49(7):1317–26. doi: 10.1038/s41366-025-01772-y (PMC12283371; doi:10.1038/s41366-025-01772-y)
Supplement: Supplementary file 1 — Supplemental information [file 41366_2025_1772_MOESM1_ESM.pdf]

Supplemental information

Data cleaning for height, weight and BMI

We used the same data cleaning strategy as that used in the Australian Longitudinal Study on Women's Health (ALSWH) [1]. Extreme values for height, weight, and BMI, and change in height and weight were audited and unless a data-entry error was identified, the value stood. For example, 2 cases had a recorded height of 205 cm (just outside of the cut-off value for extreme height measurements), but this measurement was recorded again at follow-up so the value stood as a valid measurement. For extreme weight changes (> 10% per year), we additionally consulted participant reports of whether their weight had changed in the past year. For example, one case had a weight loss of 20% in a year, but the value stood as they reported that they had indeed intentionally lost a considerable amount of weight. Extremes were defined as:

- Weight < 20kg or > 200kg

- Height < 120cm or > 200cm

- Weight loss or gain > 10% per year, with a maximum change over the whole period being 40%

- Height change > 5cm per year

- BMI < 14 or > 55

Validity of self-reported height, weight, and BMI

A subsample of participants from the Bus Santé study had height and weight data measured by trained health professionals following their completion of the self-report measures during 2023 (N=754). We therefore assessed the validity of self-reported against measured height, weight, and BMI in this subsample using Pearson's correlation, Bland–Altman plots, and Cohen's kappa statistic. Strong correlations were observed between self-reported and measured height (r=0.97; p<0.001), weight (r=0.94; p<0.001), and BMI (r=0.97; p<0.001). Bland–Altman plots indicated that the mean difference between self-reported and direct BMI measurements was small in the total sample (0.2 kg/m2). Most values fell within the limits of agreement (2 standard deviations (SD)), with random scatter plots and no systemic bias detected. The classification of BMI from self-reported and direct measurements showed that 91% were placed in the equivalent weight category with very good agreement (Cohen's kappa=0.84; 95% confidence interval (CI), 0.83-0.86).

Supplementary Table 1. An overview of measures and assessment points in the study

Time of assessment	Inclusion (2020-2021)	First follow-up (Spring 2022)	Second follow-up (Spring 2023)	Third follow-up (Spring 2024)
Behavioural factors				
Physical activity		X		
Screen time			X	
Sleep duration	X			
Fruit and vegetable consumption				X
Binge drinking		X		
Antidepressant medication use	X			
Emotional eating				X
Psychosocial factors				
Financial hardship	X			
Anxiety and depressive symptoms	X			
Loneliness	X			
Perceived stress	X			
Social support	X			
Self-esteem				X
Self-efficacy				X
Personal mastery			X	
Mental health outcomes				
Anxiety and depressive symptoms				X
Well-being				X
Body satisfaction				X
Self-rated health				X
Quality of life				X

Supplementary Table 2. Comparison of the study sample with the total sample at inclusion. Data are presented as mean (SD) for continuous measures, and % (n) for categorical measures.

	Total sample at inclusion	Study sample	p-value
	N=13,260	N=7,388	
Age at inclusion, years	47.2 (14.3)	50.7 (13.6)	<0.001
Sex			0.029
Male	42.1 (5,564)	40.6 (2,987)	
Female	57.9 (7,645)	59.4 (4,377)	
Education level			<0.001
Tertiary	63.7 (8,420)	65.2 (4,806)	
Secondary	30.8 (4,067)	31.1 (2,292)	
Primary	5.6 (737)	3.8 (277)	
Physical health condition			0.001
No	79.1 (10,490)	77.2 (5,703)	
Yes	20.9 (2,770)	22.8 (1,685)	
Frequency of moderate physical activity, 1=never, 6=every day	4.0 (1.5)	4.0 (1.5)	0.76
Frequency of vigorous physical activity, 1=never, 6=every day	2.7 (1.4)	2.7 (1.4)	0.84
Daily portions of fruit and vegetables	3.1 (1.8)	3.1 (1.8)	0.98
Binge drinking			0.72
Less than once a month	59.7 (2,991)	60.1 (2,754)	
Once a month or more	40.3 (2,018)	39.9 (1,831)	
Sleep duration per night			0.51
≥ 7 hours	68.6 (9,073)	69.1 (5,094)	
< 7 hours	31.4 (4,145)	30.9 (2,279)	
Anti-depressant drug use			0.024
No	96.9 (12,848)	96.3 (7,115)	
Yes	3.1 (412)	3.7 (273)	
Emotional eating score, range 1–5	2.4 (1.2)	2.4 (1.2)	0.97
Social support score, range 3–14	10.3 (1.9)	10.3 (1.9)	0.73
Financial difficulties			<0.001
No difficulties	53.4 (7,062)	56.7 (4,190)	
Average difficulties	29.5 (3,896)	30.2 (2,232)	
Important difficulties	7.8 (1,034)	6.6 (485)	
I don't wish to respond	9.4 (1,237)	6.5 (477)	
Self-esteem score, range 1–5	4.2 (0.7)	4.2 (0.7)	0.96
Self-efficacy score, range 1–5	4.0 (0.6)	4.0 (0.6)	0.97
Life mastery score, range 4–16	12.7 (2.9)	12.7 (2.9)	0.96
Loneliness score, range 3–9	4.6 (1.7)	4.6 (1.7)	0.12
Depressive symptoms, range 0–6	1.0 (1.1)	0.9 (1.1)	0.006
Anxiety symptoms, range 0–6	1.2 (1.2)	1.1 (1.2)	0.083
BMI	24.5 (4.9)	24.6 (4.9)	0.44
BMI category			0.61
Healthy weight	59.2 (7,828)	58.5 (4,324)	

Underweight	3.1 (410)	2.9 (214)	
Overweight	27.8 (3,685)	28.5 (2,105)	
Obesity	9.9 (1,309)	10.1 (744)	

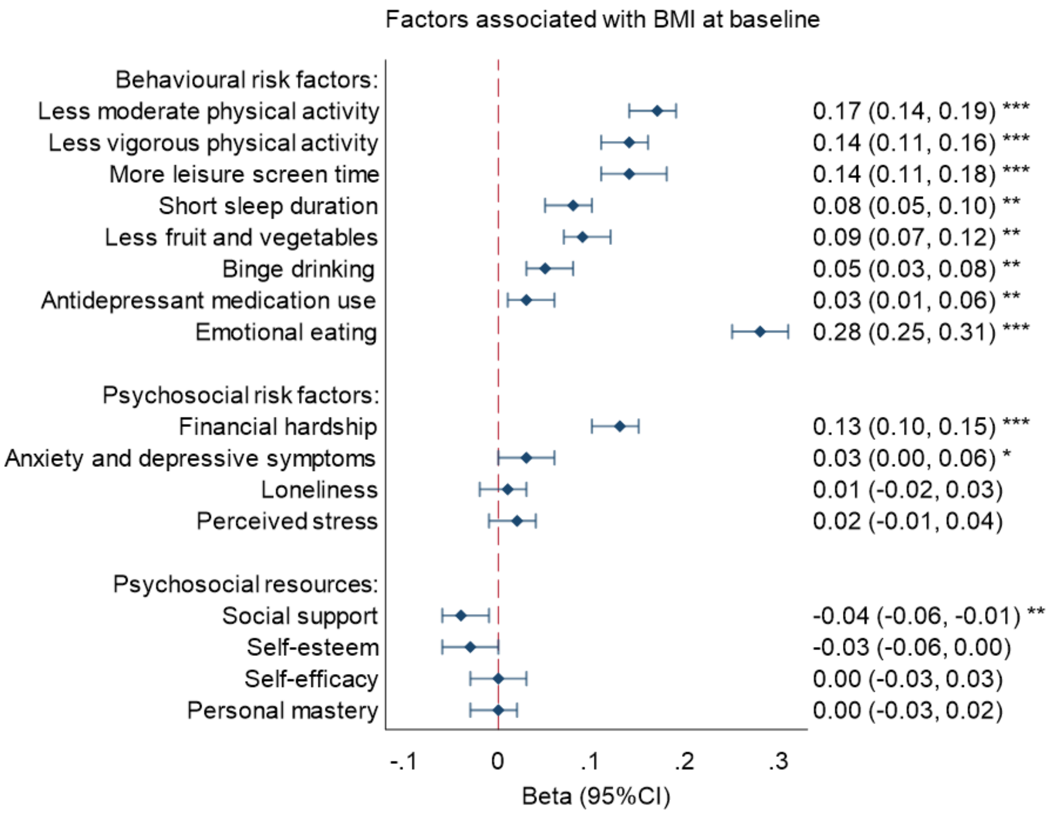

Supplementary Figure 1. Behavioural and psychosocial factors associated with BMI at baseline after adjustment for age, sex, education, and physical health condition. Results are standardised betas with 95 % confidence intervals. $*$ $p < 0.05$, $**$ $p < 0.01$, $***$ $p < 0.001$. Short sleep duration = < 7 hours. Minimum N=4313, maximum N=7388.

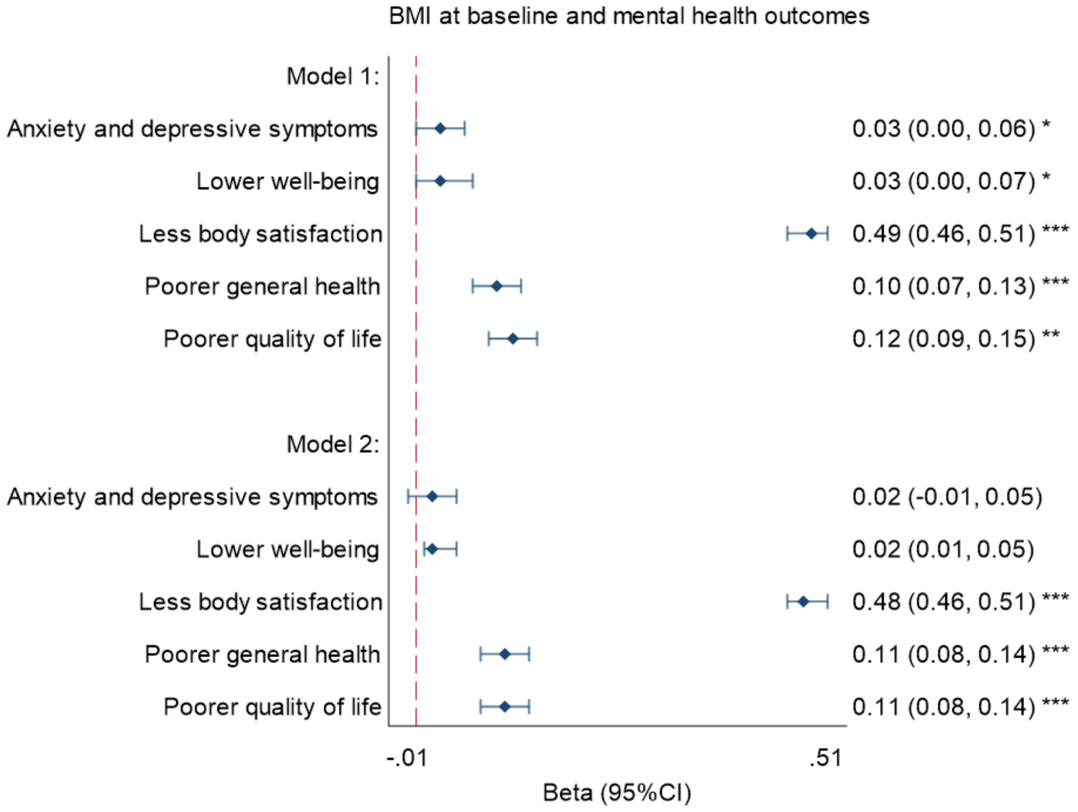

Supplementary Figure 2. Associations between BMI at baseline and mental health outcomes at follow-up (2024) after adjustment for age, sex, education, physical health condition (model 1), and anxiety and depressive symptoms at baseline (model 2). Results are standardised betas with 95 % confidence intervals. $*$ $p < 0.05$, $**$ $p < 0.01$, $***$ $p < 0.001$. N=4313.

References

1 Ball J, Ford J, Russell A, Williams L, Hockey R. Data Cleaning for Height and Weight – 1973-78 and 1946-51 cohorts. 2009. https://alswh.org.au/wp-content/uploads/2020/08/DDSSection3Data-Cleaning-for-Height-and-Weight.pdf.
